# Clinical outcomes and treatment patterns among Medicare patients with nonvalvular atrial fibrillation (NVAF) and chronic kidney disease

**DOI:** 10.1371/journal.pone.0225052

**Published:** 2019-11-14

**Authors:** Lauren E. Wilson, Xuemei Luo, Xiaoyan Li, Jack Mardekian, Alessandra B. Garcia Reeves, Asheley Skinner

**Affiliations:** 1 Department of Population Health Sciences, School of Medicine, Duke University, Durham, NC, United States of America; 2 Pfizer, Inc., New York City, NY, United States of America; 3 Bristol Myers-Squibb Company, New York City, NY, United States of America; 4 University of North Carolina at Chapel Hill, Chapel Hill, NC, United States of America; Kaohsiung Medical University Hospital, TAIWAN

## Abstract

**Background:**

Patients with nonvalvular atrial fibrillation (NVAF) and chronic kidney disease (CKD) have increased risk of adverse outcomes. This study evaluated treatment with oral anticoagulants and outcomes in elderly NVAF patients with CKD.

**Methods:**

Retrospective observational cohort study of US Medicare fee-for-service patients aged ≥66 years with comorbid CKD (advanced: Stage 4 and higher; less advanced: Stages 1–3) and a new NVAF diagnoses from 2011–2013. *A*ll-cause mortality, stroke, major bleeding, and myocardial infarction rates were estimated for 1 year post-NVAF diagnosis. Associations between CKD stage and outcomes were evaluated with multivariate-adjusted Cox regression. We assessed oral anticoagulant (OAC) receipt within 90 days post-NVAF diagnosis and associations between OAC receipt and outcomes.

**Results:**

There were 198,380 eligible patients (79,681 with advanced CKD). After adjustment for age, gender, and comorbidities, advanced CKD was associated with increased mortality (Stage 5 HR 1.47; 95% CI 1.42–1.52), MI (HR 1.48; 95% CI 1.33–1.64), stroke (HR 1.23; 95% CI 1.11–1.37) and major bleed (HR 1.44; 95% CI 1.36–1.53) risks. Among Medicare Part D enrollees who survived ≥90 days post-NVAF diagnosis, 65–71% received no OACs in the first 90 days. Those receiving warfarin (HR 0.73; 95% CI 0.71–0.75) or DOACs (HR 0.52; 95% CI 0.49–0.56) within the first 90 days had reduced mortality in the period 90 days to 1 year following NVAF diagnosis compared to those without.

**Conclusion:**

Elderly NVAF patients with advanced CKD (Stage 4 or higher) had higher mortality risks and serious clinical outcomes than those with less advanced CKD (Stage 1–3). OAC use was low across all CKD stages, but was associated with a lower mortality risk than no OAC use in the first year post-NVAF diagnosis.

## 1. Introduction

Patients with chronic kidney disease (CKD) of any severity are at higher risk for developing nonvalvular atrial fibrillation (NVAF) than the general population [1, 2]. Renal impairment is also associated with increased risk of both stroke and major bleeding in patients with NVAF, complicating therapeutic management [3–6]. Oral anticoagulation is the first-line therapy for stroke prophylaxis in NVAF, but anticoagulant treatment increases the risk of major bleeding events [4].

High-risk patients such as patients with comorbid NVAF and CKD may experience a net positive benefit of oral anticoagulation despite elevated bleeding risks [7, 8]. The most recent American Heart Association guidelines recommend use of oral anticoagulants in patients with comorbid CKD and AF with an intermediate or higher risk of stroke [9]. European registry studies suggest that patients with NVAF and CKD using warfarin had a lower risk of mortality, myocardial infarction (MI), and stroke [8, 10, 11]. Other similar studies have generated conflicting results about stroke and bleeding risk [12, 13], and a recent meta-analysis suggests that specific risk profiles may differ in patients with end-stage renal disease vs. patients with less severe CKD [14].

The approval of direct thrombin and Factor Xa inhibitors including dabigatran, rivaroxaban and apixaban for stroke prophylaxis has also introduced new therapeutic options for patients with NVAF and CKD. These direct oral anticoagulants (DOACs) are partially excreted through the renal system, so their antithrombotic effects and clinical risk-benefit ratio may differ from warfarin depending on severity of the patient’s renal impairment [15–17]. A comprehensive review of randomized clinical trials indicated that DOACs provided similar stroke prevention benefits to warfarin without increased risk of bleeding in patients with CKD stage <4 and NVAF [18]. However, these agents may increase bleeding risks in patients with NVAF where renal excretion is significantly impaired [19].

There is little real-world data on therapeutic approaches and clinical outcomes in the US elderly patient population with comorbid NVAF and CKD. The goal of this study was to provide evidence that may inform clinical management of this patient population by assessing the real-world clinical outcomes and utilization of FDA-approved oral anticoagulants for stroke prevention among elderly US patients with comorbid NVAF and CKD across a wide spectrum of kidney function.

## 2. Methods

### 2.1 Data sources and study population

This retrospective observational cohort study used research-identifiable fee-for-service (FFS) Medicare claims data including inpatient, outpatient, and carrier claims for all US Medicare beneficiaries aged 66+ with both an NVAF diagnosis in 2011–2013 and a CKD diagnosis in the 12 months (365 days) prior to the first NVAF diagnosis. Patient data was anonymized, and use of the administrative claims files for this study was approved both by the Centers for Medicare and Medicaid Services (CMS) and by the Duke University Health System Institutional Review Board. NVAF was defined as an ICD-9 CM code 427.31 in any position on an inpatient claim or on at least two outpatient or carrier claims within one year between 2011 and 2013 [20]. The first NVAF diagnosis date served as the index date. Patients were required to have continuous Medicare fee-for-service enrollment for at least 365 days prior to the index date and for 365 days after the index date or until death. All patients were required to be free of NVAF diagnoses for 12 months prior to their first NVAF diagnosis in 2011–2013. Chronic kidney disease was first broadly defined using an established claims based algorithm [21, 22] (S1 Table), and was then limited to patients with ICD-9 CM codes 585.1–585.6 indicating specific stages of CKD in any diagnosis position on an inpatient, carrier, or outpatient claim. Patients with missing or unspecified CKD stages were excluded from the analysis (S1 Fig). Advanced CKD was considered to be Stage 4 and higher; less advanced CKD was defined as Stages 1–3. Patients with valvular heart disease (S2 Table) in the 12 months prior to NVAF were also excluded.

Medicare Part D provides insurance coverage for self-administered prescription drugs such as oral anticoagulants. A sub-cohort of beneficiaries who were continuously enrolled in Medicare Part D for 12 months prior to their NVAF index date and at least 3 months afterwards are included to evaluate oral anticoagulant use in the comorbid CKD and NVAF patient population.

### 2.2 Patient demographics and comorbidities

Information on age, gender, and US geographic region of residence at the time of NVAF diagnosis was drawn from the Medicare Master Beneficiary Summary Files (MBSF). Baseline patient comorbidities, CHADS_2_, and CHADS_2_VASC stroke risk scores [23, 24] were calculated using Medicare fee-for-service inpatient, outpatient, and carrier claims from the 12 months prior to a beneficiary’s NVAF index date.

### 2.3 Oral anticoagulation treatment patterns

Patterns of treatment with oral anticoagulants that were FDA-approved for stroke prevention in NVAF patients were assessed for the sub-cohort of patients continuously enrolled in Medicare Part D during the 3 month (90 day) window after the index NVAF date and in the 12 months prior to NVAF. Medicare Part D Prescription Drug Event files were used to identify each warfarin and DOAC (apixiban, dabigatran, or rivaroxaban) prescription filled. Edoxaban was not included in this study as it was not FDA-approved until 2015 [25]. Usage was categorized both by type of oral anticoagulant received (if at all), and the timing of OAC receipt relative to the patient’s NVAF index date.

### 2.4 Clinical outcomes in the 12 months following NVAF

All-cause mortality, all-cause hospital readmission, ischemic stroke, hemorrhagic stroke, systemic embolism (SE), myocardial infarction (MI), major bleeding, and any bleeding were identified in the 12 months post-NVAF diagnosis using Medicare fee-for-service inpatient and outpatient claims data. Stroke, SE, MI, and major bleeding were identified from inpatient claims with a relevant code in the first diagnostic position (S3 Table). “Any bleeding” was defined by a code indicating GI bleeding, intracranial hemorrhage, or other bleeding based on the first or second listed ICD-9-CM diagnosis or procedure code for inpatient admissions (Inpatient file) or in any position for an outpatient encounter. All-cause mortality was ascertained from death dates in the Carrier files, and all-cause readmission was calculated using admission dates from Inpatient claims files.

### 2.5 Statistical analysis

The distribution of demographic, clinical, and treatment characteristics across the full cohort and by Medicare Part D enrollment status was estimated using proportions for binary and categorical variables and medians/interquartile ranges for continuous variables.

Using the full patient cohort, the 12 month cumulative incidence was calculated for adverse clinical events by CKD stage at the time of index NVAF diagnosis, as were unadjusted event rates per 100 person-years with death as a competing risk. The Kaplan-Meier method was used to determine the cumulative incidence of mortality, and the cumulative incidence function with competing risks was used for all other adverse outcomes. Cox proportional hazards regression was used to calculate multivariate adjusted hazard ratios for the association between CKD stage and time to the first event. Models were adjusted for patient age, gender, and baseline comorbid conditions. Death was treated as a competing risk for all outcomes except all-cause mortality.

A landmarked survival analysis was conducted to evaluate the relationship between OAC use during 3 months after NVAF diagnosis and clinical outcomes occurring 3–12 months post-NVAF among patients in the Medicare Part D sub-cohort. OAC treatment status during this 3 month time window was assessed and cumulative incidence and unadjusted event rates per 100 person-years for adverse clinical events occurring 3–12 months post-NVAF diagnosis were calculated by treatment status. Patients were categorized by treatment received during the 3 months as following: no receipt of an OAC prescription fill, receipt of a warfarin prescription fill only, and receipt of a DOAC prescription fill with or without a warfarin fill. Multivariate Cox proportional hazards regression was conducted to estimate the associations between OAC status and risk of clinical outcomes. Models were adjusted for patient CKD stage at NVAF diagnosis, age, gender, and baseline comorbid conditions. Death served as a competing risk for all outcomes except all-cause mortality.

## 3. Results

### 3.1 Cohort characteristics

A total of 198,380 patients ≥66 years old with CKD had a new NVAF diagnosis in 2011–2013 and met all eligibility criteria: 3,836 in stage 1, 12,326 in stage 2, 102,537 in stage 3, 39,230 in stage 4 and 40,451 in stage 5, including patients classified as having end stage renal disease (Table 1). This cohort of patients with both NVAF and CKD is primarily age 80 years and older (54.3%). Patients with CKD and NVAF have a significant comorbidity burden. Stroke risk was high: 47.5% had a CHADS_2_ score of 3–4 and 15.2% had a score of 5+. In the year prior to NVAF diagnosis, approximately half of the cohort had congestive heart failure, half had diabetes, one third had a major bleeding event, one third had a stroke, and one quarter had a MI.

**Table 1:** Baseline characteristics of patients stratified by CKD Stage at NVAF diagnosis (N = 198,380).

Variable	S1	S2	S3	S4	S5*	5H^1^	P-value^2^
N	3,836	12,326	102,537	39,230	5,040	35,411	
Demographics							
Age							< .001
66–74	1,056 (27.5%)	3,642 (29.5%)	26,043 (25.4%)	8,754 (22.3%)	1,335 (26.5%)	15,309 (43.2%)	
75–79	717 (18.7%)	2,495 (20.2%)	20,254 (19.8%)	7,168 (18.3%)	862 (17.1%)	7,435 (21.0%)	
80+	2,063 (53.8%)	6,189 (50.2%)	56,240 (54.8%)	23,308 (59.4%)	2,843 (56.4%)	12,667 (35.8%)	
Sex							< .001
Male	1,919 (50.0%)	6,524 (52.9%)	53,913 (52.6%)	19,119 (48.7%)	2,479 (49.2%)	19,172 (54.1%)	
Female	1,917 (50.0%)	5,802 (47.1%)	48,624 (47.4%)	20,111 (51.3%)	2,561 (50.8%)	16,239 (45.9%)	
Region of residence							< .001
Midwest	715 (18.6%)	2,822 (22.9%)	26,696 (26.0%)	10,531 (26.8%)	1,237 (24.5%)	8,382 (23.7%)	
Northeast	736 (19.2%)	2,001 (16.2%)	16,906 (16.5%)	6,752 (17.2%)	923 (18.3%)	6,149 (17.4%)	
Other/Unknown	50 (1.3%)	82 (0.7%)	596 (0.6%)	235 (0.6%)	28 (0.6%)	267 (0.8%)	
South	1,632 (42.5%)	5,297 (43.0%)	41,422 (40.4%)	15,596 (39.8%)	1,994 (39.6%)	14,451 (40.8%)	
West	703 (18.3%)	2,124 (17.2%)	16,917 (16.5%)	6,116 (15.6%)	858 (17.0%)	6,162 (17.4%)	
Charlson Comorbidity, Median (Q1, Q3)	5.0 (4.0, 7.0)	6.0 (4.0, 7.0)	6.0 (4.0, 8.0)	6.0 (4.0, 8.0)	6.0 (4.0, 8.0)	7.0 (5.0, 9.0)	< .001
CHADS2 score, Median (Q1, Q3)	3.0 (2.0, 4.0)	3.0 (2.0, 4.0)	3.0 (2.0, 4.0)	3.0 (3.0, 4.0)	3.0 (3.0, 4.0)	3.0 (3.0, 5.0)	< .001
CHADS2-VASC score, Median (Q1, Q3)	5.0 (4.0, 6.0)	5.0 (4.0, 6.0)	5.0 (4.0, 6.0)	6.0 (4.0, 7.0)	5.0 (4.0, 7.0)	6.0 (4.0, 7.0)	< .001
Prior major bleed	1,199 (31.3%)	3,743 (30.4%)	31,832 (31.0%)	12,721 (32.4%)	1,765 (35.0%)	13,238 (37.4%)	< .001
Prior stroke	1,088 (28.4%)	3,727 (30.2%)	31,679 (30.9%)	12,550 (32.0%)	1,553 (30.8%)	12,021 (33.9%)	< .001
Prior hemodialysis	11 (0.3%)	18 (0.1%)	269 (0.3%)	305 (0.8%)	146 (2.9%)	21,179 (59.8%)	< .001
Prior thrombocytopenia	310 (8.1%)	1,149 (9.3%)	10,301 (10.0%)	4,197 (10.7%)	598 (11.9%)	5,626 (15.9%)	< .001
Prior anemia	2,190 (57.1%)	6,949 (56.4%)	63,681 (62.1%)	30,045 (76.6%)	4,271 (84.7%)	33,030 (93.3%)	< .001
Prior congestive heart failure	1,625 (42.4%)	5,184 (42.1%)	47,198 (46.0%)	22,427 (57.2%)	2,933 (58.2%)	21,505 (60.7%)	< .001
Prior diabetes	1,989 (51.9%)	6,442 (52.3%)	54,719 (53.4%)	22,814 (58.2%)	3,000 (59.5%)	24,998 (70.6%)	< .001
Prior hypertension	3,649 (95.1%)	11,755 (95.4%)	98,710 (96.3%)	38,214 (97.4%)	4,924 (97.7%)	34,396 (97.1%)	< .001
Prior myocardial infarction	710 (18.5%)	2,572 (20.9%)	22,498 (21.9%)	9,536 (24.3%)	1,200 (23.8%)	9,963 (28.1%)	< .001
Prior dyspepsia or stomach discomfort	108 (2.8%)	396 (3.2%)	2,841 (2.8%)	1,061 (2.7%)	116 (2.3%)	1,070 (3.0%)	< .001
Prior peripheral vascular disease	2,469 (64.4%)	8,251 (66.9%)	70,549 (68.8%)	28,474 (72.6%)	3,539 (70.2%)	26,998 (76.2%)	< .001
Prior peripheral artery disease	825 (21.5%)	2,786 (22.6%)	23,971 (23.4%)	10,358 (26.4%)	1,277 (25.3%)	11,480 (32.4%)	< .001
Prior transient ischemic attack	264 (6.9%)	931 (7.6%)	7,076 (6.9%)	2,728 (7.0%)	281 (5.6%)	2,381 (6.7%)	< .001
Prior coronary artery disease	2,093 (54.6%)	7,060 (57.3%)	60,670 (59.2%)	24,853 (63.4%)	3,113 (61.8%)	23,360 (66.0%)	< .001

*Stage 5 chronic kidney disease without hemodialysis

^1^Stage 5 chronic kidney disease with hemodialysis

^2^P-value generated with chi-square test

Baseline patient demographic and clinical characteristics differed substantially across CKD stages at NVAF diagnosis (Table 1). Patients with end stage renal disease were most likely to be younger (66–74 years). Patients with CKD stage 4 or 5 were more likely to have serious comorbidities. S4 Table presents demographics and clinical characteristics of patients with missing or unknown CKD stages who were excluded from the cohort.

### 3.2 Treatment patterns

Of the patients in the Medicare Part D-enrolled sub-cohort who survived at least 3 months post-NVAF (N = 89,060), approximately 68% of the cohort did not fill any prescriptions for an OAC in the three months following NVAF diagnosis (Table 2), 26% filled a prescription for warfarin, and 5% filled a DOAC prescription. Since few patients (1.0%) filled prescriptions for both warfarin and a DOAC, this category was combined with those receiving a DOAC only for the following analyses on relationship between treatment status and clinical outcomes. The proportion of patients receiving warfarin post-NVAF diagnosis was consistent across CKD stages. However, use of DOACs differed across CKD stage: patients with CKD stage 1 were more likely to receive a DOAC in the 3 months post-NVAF diagnosis (~8%) than patients with CKD stage 4 or worse (<3%).

**Table 2:** CKD Stage distribution and OAC use in the 3 months after initial NVAF diagnosis in Part D-enrolled sub-cohort (N = 89,060).

Variable	Total N	No OAC use	Warfarin only	DOAC only	Warfarin and DOAC*
N	89,060	60,810 (68.2%)	23,109 (25.9%)	4,362 (4.8%)	779 (1.0%)
CKD Stage Distribution by OAC and DOAC use					
CKD Stage					
I	1,762	1,159 (65.7%)	466 (26.4%)	120 (6.8%)	>25 (>1.4%)^1^
II	5,555	3,665 (65.9%)	1,358 (24.4%)	460 (8.2%)	72 (1.3%)
III	45,577	30,128 (66.1%)	12,037 (26.4%)	2,913 (6.4%)	499 (1.1%)
IV	17,245	11,946 (69.2%)	4,506 (26.1%)	655 (3.8%)	138 (0.9%)
V without hemodialysis	>2125	1,503 (70.8%)	553 (26.1%)	54 (2.5%)	––^1^
V with hemodialysis	16,805	12,409 (73.8%)	4,189 (24.9%)	160 (0.9%)	47 (0.2%)

*Patients who filled a prescription for both a DOAC and for warfarin the 3 months following NVAF diagnosis

^1^Cell suppressed/anonymized in accordance with Medicare cell size suppression policies (cells with N<12 must be suppressed)

Receipt of OACs after NVAF diagnosis also varied depending on comorbidities: patients with prior major bleeding, anemia, thrombocytopenia, coronary artery disease, and peripheral vascular disease were more likely to receive no OAC vs. receiving warfarin or DOACs. However, among patients with prior stroke, the proportion of patients receiving no OAC treatment was similar to that of patients receiving warfarin (S5 Table).

Approximately 10% of the Part D patients received OACs prior to their NVAF diagnosis and continued filling them post-NVAF, but 22% newly initiated OACs in the 3 months post-NVAF. Most patients (65%) had no OAC prescription before or after NVAF (S6 Table).

### 3.3 Clinical events after NVAF diagnosis

During the 12 months after NVAF diagnosis in the full study cohort (N = 198,380), 33% of the patients died and 57% were re-hospitalized at least once (Table 3). Mortality rates differed greatly across CKD stage; 27.5% of patients with CKD stage 1–2 died (34.4 deaths per 100 person-years (py)), compared to 37.5% (51.1/100 py) and 43.0% (61.8 /100 py) in CKD stage 4 and 5/ESRD, respectively. The risk of mortality was highest soon after NVAF diagnosis; 68% of the observed deaths occurred in the 3 months immediately following NVAF diagnosis. Rates of hospital readmission also varied across CKD stages: 20% of patients with CKD stage 1–2 were readmitted within 30 days of NVAF diagnosis vs. 27% for patients with CKD stage 5/ESRD (S7 Table).

**Table 3:** Observed clinical event cumulative incidence, incidence per 100 person-years and multivariate adjusted hazard ratios for the association between advanced CKD stages at NVAF diagnosis and adverse events in the 12 months following NVAF diagnosis in total patient cohort (N = 198,380).

*Parameter*	Events (N)	Cumulative incidence (%)	Events/100 person-years	HR*	*P value*	*95% CI*
**All Cause Mortality**						
CKD stage						
1–2 (ref)	4455	27.5	34.4			
3	29694	28.9	36.4	0.99	0.512	0.96–1.02
4	14741	37.5	51.1	**1.20**	**<0.001**	**1.16–1.24**
5 & 5H	17408	43.0	61.8	**1.45**	**<0.001**	**1.40–1.50**
**Any hospitalization**						
1–2 (ref)	8413	52.1	101.3			
3	54583	53.2	106.2	1.01	0.330	0.99–1.03
4	23053	58.7	137.4	**1.15**	**<0.001**	**1.12–1.18**
5 & 5H	26481	65.4	187.7	**1.38**	**<0.001**	**1.35–1.42**
**Myocardial infarction**						
1–2 (ref)	468	2.9	3.7			
3	3135	3.0	3.9	1.00	0.925	0.91–1.11
4	1506	3.8	5.3	**1.22**	**<0.001**	**1.10–1.35**
5 & 5H	1859	4.6	6.8	**1.46**	**<0.001**	**1.32–1.63**
**Major bleed**						
1–2 (ref)	1560	9.6	12.8			
3	10835	10.6	14.2	1.07	0.01	1.01–1.13
4	4841	12.3	18.2	**1.25**	**<0.001**	**1.18–1.32**
5 & 5H	5540	13.7	21.7	**1.43**	**<0.001**	**1.35–1.52**
**Hemorrhagic stroke**						
1–2 (ref)	36	0.2	0.3			
3	256	0.2	0.3	1.02	0.862	0.79–1.33
4	105	0.3	0.4	1.16	0.327	0.87–1.55
5 & 5H	128	0.3	0.5	1.32	0.062	0.99–1.76
**Ischemic stroke**						
1–2 (ref)	422	2.6	3.3			
3	2800	2.7	3.5	1.04	0.448	0.94–1.15
4	1144	2.9	4.0	**1.18**	**0.005**	**1.05–1.32**
5 & 5H	965	2.4	3.5	**1.15**	**0.022**	**1.02–1.29**
**Any bleed**						
1–2 (ref)	2053	12.7	17.3			
3	13586	13.3	18.3	1.03	0.203	0.98–1.08
4	5463	13.9	20.9	**1.11**	**<0.001**	**1.05–1.17**
5 & 5H	5976	14.8	23.7	**1.17**	**<0.001**	**1.11–1.23**
**Systemic embolism**						
1–2 (ref)	24	0.1	0.2			
3	224	0.2	0.3	1.44	0.093	0.94–2.19
4	87	0.2	0.3	1.45	0.110	0.92–2.28
5 & 5H	86	0.2	0.3	1.52	0.077	0.96–2.41

*Hazard ratios adjusted for age, gender, region of residence, and comorbid conditions in the year before NVAF diagnosis

After multivariate adjustment for age, gender, geographic region, and prior comorbidities, risk of death remained substantially elevated for patients with CKD stage 4 (HR 1.20 95% CI 1.16–1.24) and CKD stage 5/ESRD (HR 1.45 95% CI 1.40–1.50) when compared to stage 1–2 patients (Table 3). Risks of individual clinical events were also associated with CKD stages; patients with Stage 5/ESRD were at elevated risk of ischemic stroke (HR 1.15 95% CI 1.02–1.29), myocardial infarction (HR 1.46 95% CI 1.32–1.63), major bleeding (HR 1.43 95% CI 1.35–1.52), and any bleeding event (HR 1.17 95% CI 1.11–1.23) vs. patients with stage 1–2. Patients with CKD Stage 4 were also at increased risk for these events compared to patients with stage 1–2 (Table 3). Major bleeding and any bleeding events were the most commonly occurring adverse events, with 12.8 major bleeds/100 py occurring in patients with CKD stage 1–2 and 21.7 major bleeds/100 py occurring in patients with CKD stage 5/ESRD. MI was the next most common event (6.8/100 py in CKD Stage 5/ESRD), followed by ischemic stroke (3.5/100 py in CKD Stage 5/ESRD).

### 3.4 Clinical events and OAC treatment status

The association between OAC treatment in the 3 months after NVAF diagnosis and clinical events occurring 3–12 months post-NVAF was evaluated. Table 4 presents clinical event frequencies, unadjusted rates and multivariate adjusted hazard ratios for patients with and without OAC treatment who were included in the Medicare Part D enrolled-sub-cohort. Of the 89,060 patients in this cohort, 18.4% died during the 3–12 months post-NVAF. After multivariate adjustment for CKD stage, age, geographic region and prior comorbidities, patients taking OACs had a lower hazard of death from any cause in the 3–12 months post-NVAF compared to those not receiving anticoagulation (Warfarin: HR 0.75 95% CI 0.72–0.78; DOAC: HR 0.55 95% CI 0.50–0.60). Warfarin and DOAC users were also at reduced risk of hospitalization, myocardial infarction, and ischemic stroke vs. patients with no OAC treatment. Warfarin users were at increased risk of major bleeding events and hemorrhagic stroke comparing to patients with no OAC treatment (Major bleed: HR 1.11, 95% CI 1.04–1.17; hemorrhagic stroke: HR 1.48, 95% 1.15–1.91). DOAC users also trended toward an increased risk for hemorrhagic stroke when compared with patients receiving no OAC (HR 1.32, 95% CI 0.80–2.19), but the confidence interval was wide likely due to lower number of DOAC users and low event counts. Patients receiving warfarin or DOAC were at an increased risk of any bleeding events (Warfarin HR 1.37 95% CI 1.31–1.44; DOAC HR 1.39 95% CI 1.26–1.52).

**Table 4:** Observed clinical event cumulative incidence, incidence per 100 person-years and multivariate adjusted hazard ratios for the association between OAC use in the 3 months post-NVAF diagnosis and adverse events occurring in the period from 3 months to 12 months post-diagnosis for patients enrolled in Medicare Part D (N = 89,060).

*Parameter*	Events (N)	Cumulative incidence	Events/100 person-years	HR*	*P value*	*95% CI*
**All-cause mortality**						
OAC Use						
No use (reference)	12520	20.9	31.5			
Warfarin only	3417	14.9	21.4	**0.75**	**<0.001**	**0.72–0.78**
DOAC +/- warfarin	474	9.3	12.9	**0.55**	**<0.001**	**0.50–0.60**
**Any hospitalization**						
No use (reference)	16080	26.9	47.6			
Warfarin only	5886	25.6	43.0	**0.93**	**<0.001**	**0.90–0.96**
DOAC +/- warfarin	1084	21.2	33.7	**0.79**	**<0.001**	**0.74–0.84**
**Myocardial infarction**						
No use (reference)	1204	2.0	3.1			
Warfarin only	384	1.6	2.4	**0.84**	**0.003**	**0.75–0.94**
DOAC +/- warfarin	50	0.9	1.4	**0.58**	**<0.001**	**0.44–0.77**
**Major bleed**						
No use (reference)	4240	7.1	11.0			
Warfarin only	1743	7.6	11.4	**1.11**	**<0.001**	**1.04–1.17**
DOAC +/- warfarin	324	6.3	9.2	1.05	0.364	0.94–1.18
**Hemorrhagic stroke**						
No use (reference)	168	0.3	0.4			
Warfarin only	96	0.4	**0.6**	**1.48**	**0.002**	**1.15–1.91**
DOAC +/- warfarin	17	0.3	0.4	1.32	0.280	0.80–2.19
**Ischemic stroke**						
No use (reference)	990	1.6	2.5			
Warfarin only	259	1.1	1.6	**0.67**	**<0.001**	**0.59–0.77**
DOAC +/- warfarin	57	1.1	1.6	**0.68**	**0.005**	**0.52–0.89**
**Any bleeding**						
No use (reference)	4767	8.0	12.5			
Warfarin only	2482	10.8	16.5	**1.37**	**<0.001**	**1.31–1.44**
DOAC +/- warfarin	514	10.1	14.9	**1.39**	**<0.001**	**1.26–1.52**
**Systemic embolism**						
No use (reference)	67	0.1	0.1			
Warfarin only	25	0.1	0.1	0.94	0.79	0.59–1.50
DOAC +/- warfarin	--^1^	0.1	0.1	1.68	0.15	0.83–3.42

*Hazard ratios adjusted for CKD stage at NVAF diagnosis, age, gender, region of residence, and comorbid conditions in the year before NVAF diagnosis

^1^Cell suppressed in accordance with Medicare cell size suppression policies (cells with N<12 must be suppressed)

A sensitivity analysis for the associations between OAC treatment in the 3 months after NVAF diagnosis and clinical events occurring 3–12 months post-NVAF was conducted by stratifying patients by CKD stage at NVAF diagnosis. When limited to patients with CKD Stages 4, 5, or end-stage renal disease (S8 Table), associations between warfarin use and clinical outcomes were consistent with those of the full Part D enrolled sub-cohort: warfarin use was associated with lower mortality rates, lower risk of hospitalization, reduced ischemic stroke risk, and increased risk of bleeding events. DOAC users were at lower risk of mortality, hospitalizations and MI, and at increased risk of bleeding events. In patients with CKD Stages 1, 2, or 3 at NVAF diagnosis (S9 Table), warfarin users had decreased risk of mortality, hospitalization, MI, and ischemic stroke, and were at increased risk of hemorrhagic stroke and bleeding events. Patients using DOACs had reduced risk of mortality, hospitalization, and MI, and were at increased risk of bleeding. A sensitivity analysis limited to patients with CKD Stage 5/ESRD only (S10 Table) also indicated generally consistent findings with those of the full Part D enrolled sub-cohort.

## 4. Discussion

In a large, retrospective observational cohort study of adults aged 66 and over enrolled in Medicare, patients who had both CKD and NVAF had substantial comorbidity burden at the time of NVAF diagnosis and a high risk of death and other adverse clinical events in the 12 months following NVAF diagnosis. Risks of all-cause mortality and other adverse clinical events were strongly associated with severity of CKD. OAC use was low across all CKD stages, but was associated with a lower all-cause mortality risk than no OAC use in the first year among patients who survived at least 3 months post-NVAF.

The substantial comorbidity burden associated with patients who have both CKD and NVAF makes it very challenging to manage this patient population. Most of these patients have comorbid cardiovascular disease and are at high risk of stroke, with 95% of the cohort having CHADS_2_ and CHADS_2_-VASc scores meeting recommendations for anticoagulation therapy. This population also has high rates of metabolic disease.

Mortality was high in the year following NVAF diagnosis for patients with both CKD and NVAF. In our study, 27.5% of patients with CKD stage 1–2 died within the year post-NVAF and the mortality rate reached 43.0% among those with CKD stage 5/ESRD. Similarly, a previous study of elderly Medicare patients with NVAF reported a 12-month mortality rate of 25%; this cohort was comprised mainly of patients without renal disease [20]. Patients with more advanced CKD had substantially higher 1-year mortality rates than the general Medicare NVAF population and when compared to patients with less severe CKD.

The majority of the observed deaths in this study occurred in the 90 days after NVAF diagnosis; this time period (6 months post-NVAF) was also identified as particularly high-risk in a recent population based study of CKD and NVAF patients in Canada [26]. For the analysis of the Medicare Part D-enrolled sub-cohort, mortality was evaluated in the period 90 days to 1 year following NVAF diagnosis as the first 90 days were used to assess OAC treatment. The mortality rates observed in the Medicare Part D sub-cohort were consequently lower than those of the full study cohort.

More severe CKD stage (Stage 4 or greater) at NVAF diagnosis was also associated with higher risk of ischemic stroke, hemorrhagic stroke, myocardial infarction, major bleeding, and all-cause hospital readmission. These findings are consistent with a growing body of evidence that poor or worsening glomerular filtration rate is associated with higher risk of mortality, stroke, systemic embolism, MI, and major bleeding in NVAF patients [4, 26–29]. Our findings indicate that the risk of adverse clinical outcomes in patients with CKD and NVAF is not only elevated for those patients receiving renal replacement therapy but also in patients at CKD stage 4 or 5 (eGFR≤30 ml/min) who were not receiving renal replacement therapy.

Utilization of FDA-approved oral anticoagulants, a standard therapy for stroke prevention in patients with NVAF, was low in this population of patients with both CKD and NVAF. More than 65% of patients did not receive any oral anticoagulation in the 3 months after their NVAF diagnosis across all CKD stages even though stroke risk was high, and the no treatment rate increased as CKD became more advanced. A recent observational study of Ottawa residents age 66+ with CKD and incident NVAF reported a similar and even lower rate of anticoagulation: 77% received no oral anticoagulation therapy in the 30 days post-discharge [30]. Oral anticoagulation is potentially underutilized in this patient population due to the observed lower long-term mortality rates in patients receiving anticoagulation and previously reported clinical risk-benefit ratios [3, 8]. Another study with longer-term follow-up post-NVAF diagnosis (median 1.44 years) has reported approximately 65% of elderly CKD and AF patients received at least 1 oral anticoagulation prescription during the entire follow-up [31]. Careful individual risk-benefit analysis based on a patient’s level of renal functioning, stroke risk, hemorrhage risk and medical history will be required when making anticoagulation decisions for patients with comorbid CKD and atrial fibrillation.

The associations between OAC use and lower mortality rates in this population of patients with CKD and NVAF are largely consistent with findings of other large population-based cohorts [10, 12, 30, 32]. However, recent meta-analyses of observational studies on oral anticoagulation in comorbid CKD and AF suggest that while patients with lower stages of CKD might benefit from OAC use, patients with ESRD showed no association between OAC use and reduced mortality or stroke risk [14, 33]. Studies also reported mixed findings for OAC use and prevention of ischemic stroke in NVAF and CKD patients, with some studies reporting a decrease in stroke risk with OAC use [10, 32, 34] while others report no association or even an increased risk of ischemic stroke [12, 30, 33]. In our study, those receiving warfarin or DOACs were less likely to die in the 90 days to 1 year after NVAF diagnosis, and were less likely to experience any hospitalization, MI, or ischemic stroke. However, those receiving warfarin or DOACs were more likely to experience major bleeding events resulting in hospitalization and hemorrhagic stroke. In the sensitivity analysis limited to patients with CKD Stage 5/ESRD, warfarin use was still associated with lower risk of mortality, ischemic stroke and MI and a higher risk of hemorrhagic stroke and bleeding.

This study is subject to some limitations. First, this study used diagnosis codes to identify CKD and staging of CKD. Although a validated algorithm was used to identify CKD and stages of CKD, there were a number of patients with missing or unspecified CKD stages, which limited our ability to further evaluate these patients. Second, while the analyses of the relationships between OAC use and clinical outcomes were adjusted for a number of potential confounders, there may be remaining confounding as claims data cannot fully capture the clinical evaluation that drove treatment decisions. Third, when evaluating the relationship between OAC use and clinical outcomes, we only evaluated outcomes in the 90 days to 1 year after NVAF diagnosis as the first 90 days were used to assess OAC treatment. In routine clinical setting, patients may start OAC treatment right after NVAF diagnosis and experience clinical outcomes during the 3 months after NVAF diagnosis. Given the current study design, effects of anticoagulation on mortality and other clinical outcomes in the first 3 months after NVAF cannot be estimated from this study. Additionally, since oral anticoagulation use was only assessed in the first 3 months post-NVAF diagnosis, we likely miss patients who started on oral anticoagulants after that time period. We also did not capture use of alternate anticoagulation strategies that are not FDA-approved for stroke prevention in NVAF patients, such as use of low molecular-weight heparin. Finally, the number of patients receiving DOACs was relatively small because DOACs were newly approved during this study period.

A major strength of this study is that the population is based on a 100% sample of individuals with Medicare who were diagnosed with atrial fibrillation from 2011–2013. Since Medicare enrollment is nearly universal among the US population age 65 and older, current study findings should be generalizable to the US elderly population with NVAF and CKD that received a staging diagnosis.

## 5. Conclusion

Elderly patients with NVAF and CKD had many comorbid conditions across all CKD stages. NVAF patients with advanced CKD had higher risks of mortality and serious clinical outcomes than did patients with less advanced CKD. Use of OACs in the first 3 months following NVAF diagnosis was low, but patients surviving at least 3 months post NVAF-diagnosis who received OACs had lower subsequent mortality rates than those who did not receive OACs. Additional studies are needed to evaluate the impacts of anticoagulation therapy in the 3 months immediately following NVAF diagnosis, as well as individual OACs’ impacts on mortality and clinical outcomes in patients with NVAF and CKD including ESRD.

## Supporting information

S1 Fig Sample size flow diagram. (PDF)

S1 Table ICD-9 CM code definitions for Medicare claims-based identification of chronic kidney disease and ICD-9 codes for CKD stage assignment. (PDF)

S2 Table ICD-9-CM and CPT-4 codes for Medicare claims-based identification of patients with valvular heart disease. These patients were excluded from our analysis.
(PDF)

S3 Table ICD-9 CM code definitions for Medicare claims-based identification of clinical outcomes of interest. (PDF)

S4 Table Baseline characteristics of patients by CKD stage including patients with no CKD Stage. Unknown staging includes patients with CKD Stage coding as unknown (ICD-9 code 585.9) and patients with codes fitting the broad definition of CKD without ICD-9 codes for a specific CKD stage (N = 369,323).
(XLSX)

S5 Table Baseline demographic and clinical characteristics of patients enrolled in Medicare Part D for 12 months prior and 3 months post-NVAF by receipt of OAC (N = 89,060). (DOCX)

S6 Table Distribution of patients using an oral anticoagulant in the 12 months before and the 3 months after their NVAF diagnosis stratified by CKD stage (N = 89,060). (DOCX)

S7 Table Observed cumulative incidence for 30 day hospital readmission and multivariate adjusted hazard ratios for the association between advanced CKD stages at NVAF diagnosis and 30 day hospital readmission immediately following NVAF diagnosis in total patient cohort (N = 198,380). (DOCX)

S8 Table Sensitivity analysis. Limited to patients with CKD Stage 4, 5, or ESRD at NVAF diagnosis.: Observed clinical event cumulative incidence, incidence per 100 person-years and multivariate adjusted hazard ratios for the association between OAC use in the 3 months post-NVAF diagnosis and adverse events occurring in the period from 3 months post-diagnosis to 12 months post-diagnosis for patients enrolled in Medicare Part D.
(DOCX)

S9 Table Sensitivity analysis. Limited to patients with CKD Stage 1, 2, or 3 at NVAF diagnosis. Observed clinical event cumulative incidence, incidence per 100 person-years and multivariate adjusted hazard ratios for the association between OAC use in the 3 months post-NVAF diagnosis and adverse events occurring in the period from 3 months post-diagnosis to 12 months post-diagnosis for patients enrolled in Medicare Part D.
(DOCX)

S10 Table Sensitivity analysis. Limited to patients with CKD Stage 5/ESRD at NVAF diagnosis. Multivariate adjusted hazard ratios for the association between OAC use in the 3 months post-NVAF diagnosis and adverse events occurring in the period from 3 months post-diagnosis to 12 months post-diagnosis for patients enrolled in Medicare Part D (Total cohort N = 18,925: 13,912 non-users, 4,742 warfarin users, and 217 DOAC +/- warfarin users).
(DOCX)
