# Supplementary figures and images for: Clinical outcomes and treatment patterns among Medicare patients with nonvalvular atrial fibrillation (NVAF) and chronic kidney disease
Source: PLoS One. 2019 Nov 14;14(11):e0225052. doi: 10.1371/journal.pone.0225052 (PMC6855694; doi:10.1371/journal.pone.0225052)

*Supplemental Figure 1. Sample size flow diagram*

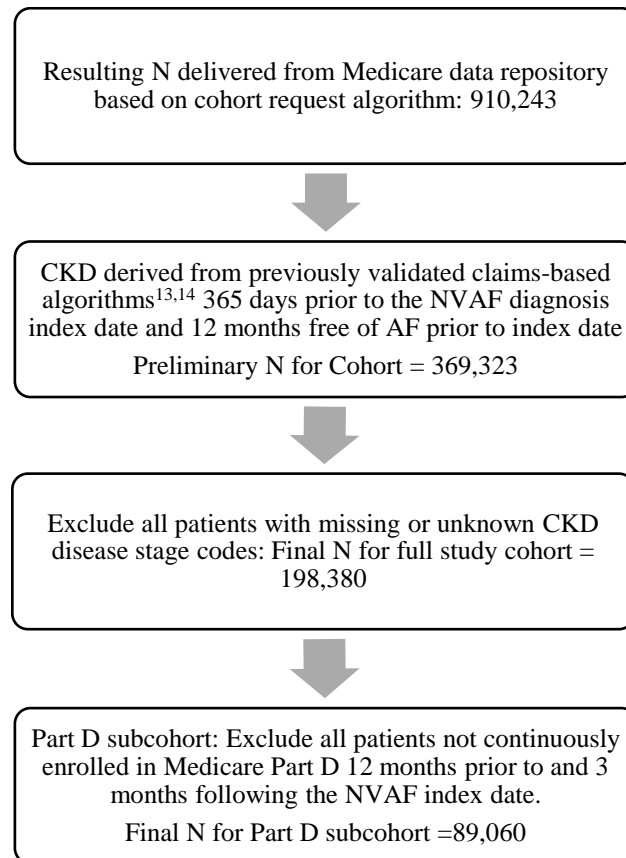

Supplement: S1 Fig — (PDF) [file pone.0225052.s001.pdf]
