# Supplementary material for: Clinical outcomes and treatment patterns among Medicare patients with nonvalvular atrial fibrillation (NVAF) and chronic kidney disease
Source: PLoS One. 2019 Nov 14;14(11):e0225052. doi: 10.1371/journal.pone.0225052 (PMC6855694; doi:10.1371/journal.pone.0225052)
Supplement: S1 Table — (PDF) [file pone.0225052.s002.pdf]

Supplemental Table 1: ICD-9 CM code definitions for Medicare claims-based identification of chronic kidney disease and ICD-9 codes for CKD stage assignment

**ICD-9 CM Code**	**Description**
016.00, 016.01, 016.02, 016.03, 016.04, 016.05, 016.06, 095.4, 189.0, 189.9, 223.0, 236.91, 249.40, 249.41, 250.40, 250.41, 250.42, 250.43, 271.4, 274.10, 283.11, 403.01, 403.11, 403.91, 404.02, 404.03, 404.12, 404.13, 404.92, 404.93, 440.1, 442.1, 572.4, 580.0, 580.4, 580.81, 580.89, 580.9, 581.0, 581.1, 581.2, 581.3, 581.81, 581.89, 581.9, 582.0, 582.1, 582.2, 582.4, 582.81, 582.89, 582.9, 583.0, 583.1, 583.2, 583.4, 583.6, 583.7, 583.81, 583.89, 583.9, 584.5, 584.6, 584.7, 584.8, 584.9, 585.1, 585.2, 585.3, 585.4, 585.5, 585.6, 585.9, 586, 587, 588.0, 588.1, 588.81, 588.89, 588.9, 591, 753.12, 753.13, 753.14, 753.15, 753.16, 753.17, 753.19, 753.20, 753.21, 753.22, 753.23, 753.29, 794.4	From inpatient carrier and outpatient claim in any position.
585.1	Stage I CKD
585.2	Stage II CKD
585.3	Stage III CKD
585.4	Stage IV CKD
585.5, 585.6	Stage V CKD
585.9	Stage unspecified
