# Supplementary material for: Clinical outcomes and treatment patterns among Medicare patients with nonvalvular atrial fibrillation (NVAF) and chronic kidney disease
Source: PLoS One. 2019 Nov 14;14(11):e0225052. doi: 10.1371/journal.pone.0225052 (PMC6855694; doi:10.1371/journal.pone.0225052)
Supplement: S2 Table — These patients were excluded from our analysis. (PDF) [file pone.0225052.s003.pdf]

Supplemental Table 2: ICD-9-CM and CPT-4 codes for Medicare claims-based identification of patients with valvular heart disease. These patients were excluded from our analysis.

Code	Description
**ICD-9-CM:** 394, 394.0, 394.1, 394.2, 394.9, 395, 395.0, 395.1, 395.2, 395.9, 396, 396.0, 396.1, 396.2, 396.3, 396.8, 398.9, 397, 397.0, 397.1, 397.9, 424.0, 424.1, 424.2, 424.3, 424.9, 424.90, 424.91, 424.99	Valvular disease diagnosis from inpatient, carrier and outpatient claim in any position.
**ICD-9-CM:** 35.00, 35.01, 35.02, 35.03, 35.04, 35.10, 35.11, 35.12, 35.13, 35.14, 35.20, 35.21, 35.22, 35.23, 35.24, 35.25, 35.26, 35.27, 35.28<br><br>**CPT-4:** 33400, 33401, 33402, 33403, 33404, 33405, 33406, 33407, 33408, 33409, 33410, 33411, 33412, 33413; 33420, 33421, 33422, 33423, 33424, 33425, 33426, 33427, 33428, 33429, 33430, 33460, 33461, 33462, 33463, 33464, 33465, 33466, 33467, 33468, 33469, 33470, 33471, 33472, 33473, 33474, 33475	Procedure code for valve surgery from inpatient, carrier and outpatient claims in any position
