# Supplementary material for: Clinical outcomes and treatment patterns among Medicare patients with nonvalvular atrial fibrillation (NVAF) and chronic kidney disease
Source: PLoS One. 2019 Nov 14;14(11):e0225052. doi: 10.1371/journal.pone.0225052 (PMC6855694; doi:10.1371/journal.pone.0225052)
Supplement: S3 Table — (PDF) [file pone.0225052.s004.pdf]

Supplemental Table 3: ICD-9 CM code definitions for Medicare claims-based identification of clinical outcomes of interest

**ICD-9 CM Code**	**Description**	**Outcome**
433.x1, 434.x1, 436.xx	From inpatient claim in first position.	Ischemic stroke
430.xx-432.xx<br><br>Cases will excluded if traumatic brain injury (ICD-9: 800-804, 850-854) was present during hospitalization.	From inpatient claim in first position.	Hemorrhagic stro[illegible]
444.x, 445.x	From inpatient claim in first position.	Systemic embolis[illegible]
456.0x, 456.20, 530.82, 531.0x, 531.2x, 531.4x, 531.6x, 532.0x, 532.2x, 532.4x, 532.6x, 533.0x, 533.2x, 533.4x, 533.6x, 534.0x, 534.2x, 534.4x, 534.6x, 535.01, 535.11, 535.21, 535.31, 535.41, 535.51, 535.61, 537.83, 562.02, 562.03, 562.12, 562.13, 568.81, 569.3, 569.85, 578.x, 430.xx, 431.xx, 432.0x, 432.1x, 432.9x, 852.0x, 852.2x, 852.4x, 853.0x, 285.1, 360.43, 362.43, 362.81, 363.61, 363.62, 363.72, 364.41, 372.72, 374.81, 376.32, 377.42, 379.23, 423.0x, 596.7x, 599.7x, 602.1x, 620.1, 621.4, 626.2, 626.5, 626.7, 626.8, 626.9, 719.1x, 782.7, 784.7, 784.8, 786.3x, 958.2, 997.02, 998.11<br>**Procedure codes:** 99.04, 44.43	From inpatient claim in first position.	Major bleeding
410.xx; 412.xx	From inpatient claim in first position.	Myocardial infarc[illegible]
456.0x, 456.20, 530.82, 531.0x, 531.2x, 531.4x, 531.6x, 532.0x, 532.2x, 532.4x, 532.6x, 533.0x, 533.2x, 533.4x, 533.6x, 534.0x, 534.2x, 534.4x, 534.6x, 535.01, 535.11, 535.21, 535.31, 535.41, 535.51, 535.61, 537.83, 562.02, 562.03, 562.12, 562.13, 568.81, 569.3, 569.85, 578.x, 430.xx, 431.xx, 432.0x, 432.1x, 432.9x, 852.0x, 852.2x, 852.4x, 853.0x, 285.1, 360.43, 362.43, 362.81, 363.61, 363.62, 363.72, 364.41, 372.72, 374.81, 376.32, 377.42, 379.23, 423.0x, 596.7x, 599.7x, 602.1x, 620.1, 621.4, 626.2, 626.5, 626.7, 626.8, 626.9, 719.1x, 782.7, 784.7, 784.8, 786.3x, 958.2, 997.02, 998.11<br>**Procedure codes:** 99.04, 44.43	first or second listed diagnosis or procedure code for inpatient claim or any position for outpatient claim	Any bleeding
