# Supplementary material for: Clinical outcomes and treatment patterns among Medicare patients with nonvalvular atrial fibrillation (NVAF) and chronic kidney disease
Source: PLoS One. 2019 Nov 14;14(11):e0225052. doi: 10.1371/journal.pone.0225052 (PMC6855694; doi:10.1371/journal.pone.0225052)
Supplement: S5 Table — (DOCX) [file pone.0225052.s006.docx]

***Supplemental Table 5****:* Baseline demographic and clinical characteristics of patients enrolled in Medicare Part D for 12 months prior and 3 months post-NVAF by receipt of OAC (N=89,060)

| **Variable** | **No use** | **Warfarin only** | **DOAC only** | **Warfarin/DOAC** | **p-value** |
| --- | --- | --- | --- | --- | --- |
| N | 60,810 | 23,109 | 4,362 | 779 |  |
|  |  |  |  |  |  |
| Demographics by OAC and DOAC use |  |  |  |  |  |
| Age |  |  |  |  | < .001 |
| 66-74 | 18,543 (30.5%) | 8,359 (36.2%) | 1,480 (33.9%) | 294 (37.7%) |  |
| 75-79 | 11,563 (19.0%) | 5,047 (21.8%) | 1,029 (23.6%) | 179 (23.0%) |  |
| 80+ | 30,704 (50.5%) | 9,703 (42.0%) | 1,853 (42.5%) | 306 (39.3%) |  |
| Sex |  |  |  |  | < .001 |
| Male | 27,366 (45.0%) | 10,710 (46.3%) | 2,050 (47.0%) | 346 (44.4%) |  |
| Female | 33,444 (55.0%) | 12,399 (53.7%) | 2,312 (53.0%) | 433 (55.6%) |  |
| Region of residence |  |  |  |  | < .001 |
| Midwest | 14,676 (24.1%) | 6,531 (28.3%) | 976 (22.4%) | 193 (24.8%) |  |
| Northeast | 10,283 (16.9%) | 4,254 (18.4%) | 642 (14.7%) | 128 (16.4%) |  |
| Other/Unknown | 321 (0.5%) | 143 (0.6%) | 26 (0.6%) | ­- |  |
| South | 25,028 (41.2%) | 8,797 (38.1%) | 1,984 (45.5%) | 319 (40.9%) |  |
| West | 10,502 (17.3%) | 3,384 (14.6%) | 734 (16.8%) | 136 (17.5%) |  |
| Charlson Comorbidity, Median (Q1, Q3) | 6.0 (4.0, 8.0) | 6.0 (4.0, 8.0) | 5.0 (4.0, 7.0) | 5.0 (4.0, 7.0) | < .001 |
| CHADS2 score, Median (Q1, Q3) | 3.0 (2.0, 4.0) | 3.0 (2.0, 4.0) | 3.0 (2.0, 4.0) | 3.0 (2.0, 4.0) | < .001 |
| CHADS2-VASC score, Median (Q1, Q3) | 5.0 (4.0, 7.0) | 5.0 (4.0, 7.0) | 5.0 (4.0, 6.0) | 5.0 (4.0, 6.0) | < .001 |
| Prior major bleed |  |  |  |  | < .001 |
| Yes | 20,027 (32.9%) | 6,361 (27.5%) | 961 (22.0%) | 171 (22.0%) |  |
| Prior stroke |  |  |  |  | < .001 |
| Yes | 19,512 (32.1%) | 7,258 (31.4%) | 1,240 (28.4%) | 206 (26.4%) |  |
| Prior hemodialysis |  |  |  |  | < .001 |
| Yes | 8,056 (13.2%) | 2,680 (11.6%) | 38 (0.9%) | 20 (2.6%) |  |
| Prior thrombocytopenia |  |  |  |  | < .001 |
| Yes | 6,322 (10.4%) | 1,893 (8.2%) | 231 (5.3%) | 54 (6.9%) |  |
| Prior anemia |  |  |  |  | < .001 |
| Yes | 44,650 (73.4%) | 15,423 (66.7%) | 2,351 (53.9%) | 439 (56.4%) |  |
| Prior congestive heart failure |  |  |  |  | < .001 |
| Yes | 31,562 (51.9%) | 11,324 (49.0%) | 1,687 (38.7%) | 325 (41.7%) |  |
| Prior diabetes |  |  |  |  | < .001 |
| Yes | 36,466 (60.0%) | 14,254 (61.7%) | 2,478 (56.8%) | 442 (56.7%) |  |
| Prior hypertension |  |  |  |  | .08 |
| Yes | 59,020 (97.1%) | 22,397 (96.9%) | 4,259 (97.6%) | 757 (97.2%) |  |
| Prior myocardial infarction |  |  |  |  | < .001 |
| Yes | 14,080 (23.2%) | 4,701 (20.3%) | 674 (15.5%) | 125 (16.0%) |  |
| Prior dyspepsia or stomach discomfort |  |  |  |  | .001 |
| Yes | 1,961 (3.2%) | 634 (2.7%) | 118 (2.7%) | 20 (2.6%) |  |
| Prior peripheral vascular disease |  |  |  |  | < .001 |
| Yes | 44,040 (72.4%) | 16,028 (69.4%) | 2,804 (64.3%) | 500 (64.2%) |  |
| Prior peripheral artery disease |  |  |  |  | < .001 |
| Yes | 16,281 (26.8%) | 6,031 (26.1%) | 891 (20.4%) | 163 (20.9%) |  |
| Prior transient ischemic attack |  |  |  |  | < .001 |
| Yes | 4,170 (6.9%) | 1,664 (7.2%) | 371 (8.5%) | 40 (5.1%) |  |
| Prior coronary artery disease |  |  |  |  | < .001 |
| Yes | 37,911 (62.3%) | 13,551 (58.6%) | 2,373 (54.4%) | 432 (55.5%) |  |
