# Supplementary material for: Clinical outcomes and treatment patterns among Medicare patients with nonvalvular atrial fibrillation (NVAF) and chronic kidney disease
Source: PLoS One. 2019 Nov 14;14(11):e0225052. doi: 10.1371/journal.pone.0225052 (PMC6855694; doi:10.1371/journal.pone.0225052)
Supplement: S6 Table — (DOCX) [file pone.0225052.s007.docx]

***Supplemental Table 6:*** Distribution of patients using an oral anticoagulant in the 12 months before and the 3 months after their NVAF diagnosis stratified by CKD stage (N=89,060)

| **Variable** | **No use pre- or post-NVAF** | **Pre-NVAF use only** | **Post-NVAF use only** | **Pre- and post-NVAF use** |
| --- | --- | --- | --- | --- |
| N | 58,016 (65.1%) | 2,794 (3.1%) | 19,449 (21.8%) | 8,801 (9.8%) |
| CKD Stage |  |  |  |  |
| I | 1,106 (62.7%) | 53 (3.0%) | 421 (23.9%) | 182 (10.3%) |
| II | 3,487 (62.7%) | 178 (3.2%) | 1,391 (25.0%) | 499 (8.9%) |
| III | 28,733 (63.0%) | 1,395 (3.0%) | 10,780 (23.6%) | 4,669 (10.2%) |
| IV | 11,381 (66.0%) | 565 (3.2%) | 3,529 (20.4%) | 1,770 (10.3%) |
| V without hemodialysis | 1,443 (68.1%) | 60 (2.8%) | 416 (19.7%) | 197 (9.3%) |
| V with hemodialysis | 11,866 (70.6%) | 543 (3.2%) | 2,912 (17.3%) | 1,484 (8.8%) |
