# Supplementary material for: Clinical outcomes and treatment patterns among Medicare patients with nonvalvular atrial fibrillation (NVAF) and chronic kidney disease
Source: PLoS One. 2019 Nov 14;14(11):e0225052. doi: 10.1371/journal.pone.0225052 (PMC6855694; doi:10.1371/journal.pone.0225052)
Supplement: S7 Table — (DOCX) [file pone.0225052.s008.docx]

***Supplemental Table 7:*** Observed cumulative incidence for 30 day hospital readmission and multivariate adjusted hazard ratios for the association between advanced CKD stages at NVAF diagnosis and 30 day hospital readmission immediately following NVAF diagnosis in total patient cohort (N=**198,380**)

| *Parameter* | Cumulative incidence (%) | *HR** | *P value* | *95% CI* |
| --- | --- | --- | --- | --- |
| **30 day hospital readmission** |  |  |  |  |
| CKD stage |  |  |  |  |
| 1-2 (ref) | 20.2 |  |  |  |
| 3 | 20.8 | 1.01 | 0.548 | 0.98-1.04 |
| 4 | 23.5 | **1.07** | **<0.001** | **1.03-1.10** |
| 5 & 5H | 27.0 | **1.14** | **<0.001** | **1.10-1.18** |
