# Supplementary material for: Clinical outcomes and treatment patterns among Medicare patients with nonvalvular atrial fibrillation (NVAF) and chronic kidney disease
Source: PLoS One. 2019 Nov 14;14(11):e0225052. doi: 10.1371/journal.pone.0225052 (PMC6855694; doi:10.1371/journal.pone.0225052)
Supplement: S9 Table — Limited to patients with CKD Stage 1, 2, or 3 at NVAF diagnosis. Observed clinical event cumulative incidence, incidence per 100 person-years and multivariate adjusted hazard ratios for the association between OAC use in the 3 months post-NVAF diagnosis and adverse events occurring in the period from 3 months post-diagnosis to 12 months post-diagnosis for patients enrolled in Medicare Part D. (DOCX) [file pone.0225052.s010.docx]

***Supplemental Table 9***: Sensitivity analysis limited to patients with CKD Stages 1, 2, or 3 at NVAF diagnosis. Multivariate adjusted hazard ratios for the association between OAC use in the 3 months post-NVAF diagnosis and adverse events occurring in the period from 3 months post-diagnosis to 12 months post-diagnosis for patients enrolled in Medicare Part D (Total cohort N=52,275)

| *Outcome* | Events (N) | Cumulative incidence (%) | Events/100 person-years | *HR** | *P value* | *95% CI* |
| --- | --- | --- | --- | --- | --- | --- |
| **All-cause mortality** |  |  |  |  |  |  |
| OAC Use |  |  |  |  |  |  |
| No use (reference) | 6080 | 17.6 | 26.0 |  |  |  |
| Warfarin only | 1647 | 11.9 | 16.9 | 0.72 | **<0.001** | 0.68-0.76 |
| DOAC +/- warfarin | 350 | 8.6 | 11.9 | 0.57 | **<0.001** | 0.51-0.63 |
| **Any hospitalization** |  |  |  |  |  |  |
| No use (reference) | 8731 | 25.3 | 43.4 |  |  |  |
| Warfarin only | 3319 | 24.1 | 39.2 | 0.93 | **<0.001** | 0.89-0.97 |
| DOAC +/- warfarin | 852 | 20.9 | 33.2 | 0.82 | **<0.001** | 0.76-0.88 |
| **Myocardial infarction** |  |  |  |  |  |  |
| No use (reference) | 565 | 1.6 | 2.4 |  |  |  |
| Warfarin only | 188 | 1.4 | 1.9 | 0.82 | **0.021** | 0.70-0.97 |
| DOAC +/- warfarin | 38 | 0.9 | 1.3 | 0.61 | **0.003** | 0.44-0.85 |
| **Major bleed** |  |  |  |  |  |  |
| No use (reference) | 2076 | 6.0 | 9.1 |  |  |  |
| Warfarin only | 872 | 6.3 | 9.2 | 1.08 | 0.075 | 0.99-1.16 |
| DOAC +/- warfarin | 252 | 6.2 | 8.9 | 1.12 | 0.089 | 0.98-1.28 |
| **Hemorrhagic stroke** |  |  |  |  |  |  |
| No use (reference) | 85 | 0.2 | 0.4 |  |  |  |
| Warfarin only | 54 | 0.4 | 0.6 | 1.57 | **0.011** | 1.11-2.21 |
| DOAC +/- warfarin | 13 | 0.3 | 0.4 | 1.35 | 0.315 | 0.75-2.44 |
| **Ischemic stroke** |  |  |  |  |  |  |
| No use (reference) | 529 | 1.5 | 2.3 |  |  |  |
| Warfarin only | 158 | 1.1 | 1.6 | 0.74 | **0.001** | 0.62-0.89 |
| DOAC +/- warfarin | 43 | 1.0 | 1.5 | 0.69 | 0.019 | 0.5-0.94 |
| **Any bleeding** |  |  |  |  |  |  |
| No use (reference) | 2558 | 7.4 | 11.4 |  |  |  |
| Warfarin only | 1372 | 9.9 | 14.9 | 1.35 | **<0.001** | 1.26-1.44 |
| DOAC +/- warfarin | 403 | 9.0 | 14.6 | 1.42 | **<0.001** | 1.28-1.58 |
| **Systemic embolism** |  |  |  |  |  |  |
| No use (reference) | 36 | 0.1 | 0.1 |  |  |  |
| Warfarin only | 13 | <0.1 | 0.1 | 0.87 | 0.673 | 0.46-1.65 |
| DOAC +/- warfarin | -^1^ | 0.1 | 0.2 | 1.67 | 0.220 | 0.74-3.79 |

*Hazard ratios adjusted for CKD stage at NVAF diagnosis, age, gender, region of residence, and comorbid conditions in the year before NVAF diagnosis

^1^Cell suppressed in accordance with Medicare cell size suppression policies (cells with N<12 must be suppressed)
