# Supplementary material for: Clinical outcomes and treatment patterns among Medicare patients with nonvalvular atrial fibrillation (NVAF) and chronic kidney disease
Source: PLoS One. 2019 Nov 14;14(11):e0225052. doi: 10.1371/journal.pone.0225052 (PMC6855694; doi:10.1371/journal.pone.0225052)
Supplement: S10 Table — Limited to patients with CKD Stage 5/ESRD at NVAF diagnosis. Multivariate adjusted hazard ratios for the association between OAC use in the 3 months post-NVAF diagnosis and adverse events occurring in the period from 3 months post-diagnosis to 12 months post-diagnosis for patients enrolled in Medicare Part D (Total cohort N = 18,925: 13,912 non-users, 4,742 warfarin users, and 217 DOAC +/- warfarin users). (DOCX) [file pone.0225052.s011.docx]

***Supplemental Table 10***: Sensitivity analysis limited to patients with CKD Stage 5/ESRD at NVAF diagnosis. Multivariate adjusted hazard ratios for the association between OAC use in the 3 months post-NVAF diagnosis and adverse events occurring in the period from 3 months post-diagnosis to 12 months post-diagnosis for patients enrolled in Medicare Part D (Total cohort N=18,925: 13,912 non-users, 4,742 warfarin users, and 217 DOAC +/- warfarin users

| *Outcome* | *HR** | *P value* | *95% CI* |
| --- | --- | --- | --- |
| **All-cause mortality** |  |  |  |
| OAC Use |  |  |  |
| No use (reference) |  |  |  |
| Warfarin only | **0.82** | **<0.001** | **0.76-0.87** |
| DOAC +/- warfarin | **0.55** | **<0.001** | **0.39-0.76** |
| **Any hospitalization** |  |  |  |
| No use (reference) |  |  |  |
| Warfarin only | 0.95 | 0.134 | 0.9-1.01 |
| DOAC +/- warfarin | 0.83 | 0.115 | 0.65-1.05 |
| **Myocardial infarction** |  |  |  |
| No use (reference) |  |  |  |
| Warfarin only | **0.80** | **0.046** | **0.65-1.00** |
| DOAC +/- warfarin | 1.15 | 0.691 | 0.57-2.33 |
| **Major bleed** |  |  |  |
| No use (reference) |  |  |  |
| Warfarin only | **1.16** | **0.006** | **1.04-1.28** |
| DOAC +/- warfarin | 0.67 | 0.114 | 0.41-1.1 |
| **Hemorrhagic stroke** |  |  |  |
| No use (reference) |  |  |  |
| Warfarin only | **1.61** | **0.047** | **1.01-2.58** |
| DOAC +/- warfarin | 1.14 | 0.898 | 0.16-8.3 |
| **Ischemic stroke** |  |  |  |
| No use (reference) |  |  |  |
| Warfarin only | **0.61** | **0.001** | **0.45-0.82** |
| DOAC +/- warfarin | 0.42 | 0.221 | 0.1-1.69 |
| **Any bleeding** |  |  |  |
| No use (reference) |  |  |  |
| Warfarin only | **1.40** | **0.000** | **1.27-1.54** |
| DOAC +/- warfarin | 1.24 | 0.247 | 0.86-1.78 |
| **Systemic embolism** |  |  |  |
| No use (reference) |  |  |  |
| Warfarin only | 1.28 | 0.547 | 0.57-2.87 |
| DOAC +/- warfarin | -^1^ | - | - |

*Hazard ratios adjusted for CKD stage at NVAF diagnosis, age, gender, region of residence, and comorbid conditions in the year before NVAF diagnosis

^1^Cell suppressed in accordance with Medicare cell size suppression policies (cells with N<12 must be suppressed)
